# *Burkholderia pseudomallei* Is Spatially Distributed in Soil in Northeast Thailand

**DOI:** 10.1371/journal.pntd.0000694

**Published:** 2010-06-01

**Authors:** Direk Limmathurotsakul, Vanaporn Wuthiekanun, Narisara Chantratita, Gumphol Wongsuvan, Premjit Amornchai, Nicholas P. J. Day, Sharon J. Peacock

**Affiliations:** 1 Department of Tropical Hygiene, Faculty of Tropical Medicine, Mahidol University, Bangkok, Thailand; 2 Mahidol-Oxford Tropical Medicine Research Unit, Faculty of Tropical Medicine, Mahidol University, Bangkok, Thailand; 3 Department of Microbiology and Immunology, Faculty of Tropical Medicine, Mahidol University, Bangkok, Thailand; 4 Center for Clinical Vaccinology and Tropical Medicine, Nuffield Department of Clinical Medicine, University of Oxford, Churchill Hospital, Oxford, United Kingdom; 5 Department of Medicine, Cambridge University, Addenbrooke's Hospital, Cambridge, United Kingdom; University of Oklahoma Health Sciences Center, United States of America

## Abstract

**Background:**

Melioidosis is a frequently fatal infectious disease caused by the soil dwelling Gram-negative bacterium *Burkholderia pseudomallei*. Environmental sampling is important to identify geographical distribution of the organism and related risk of infection to humans and livestock. The aim of this study was to evaluate spatial distribution of *B. pseudomallei* in soil and consider the implications of this for soil sampling strategies.

**Methods and Findings:**

A fixed-interval sampling strategy was used as the basis for detection and quantitation by culture of *B. pseudomallei* in soil in two environmental sites (disused land covered with low-lying scrub and rice field) in northeast Thailand. Semivariogram and indicator semivariogram were used to evaluate the distribution of *B. pseudomallei* and its relationship with range between sampling points. *B. pseudomallei* was present on culture of 80/100 sampling points taken from the disused land and 28/100 sampling points from the rice field. The median *B. pseudomallei* cfu/gram from positive sampling points was 378 and 700 for the disused land and the rice field, respectively (p = 0.17). Spatial autocorrelation of *B. pseudomallei* was present, in that samples taken from areas adjacent to sampling points that were culture positive (negative) for *B. pseudomallei* were also likely to be culture positive (negative), and samples taken from areas adjacent to sampling points with a high (low) *B. pseudomallei* count were also likely to yield a high (low) count. Ranges of spatial autocorrelation in quantitative *B. pseudomallei* count were 11.4 meters in the disused land and 7.6 meters in the rice field.

**Conclusions:**

We discuss the implications of the uneven distribution of *B. pseudomallei* in soil for future environmental studies, and describe a range of established geostatistical sampling approaches that would be suitable for the study of *B. pseudomallei* that take account of our findings.

## Author Summary

Melioidosis is a severe infection caused by the environmental bacterium *Burkholderia pseudomallei*. Soil sampling is important to identify geographic regions where humans and animals are at risk of exposure. The purpose of this study was to examine a factor that has a major bearing on the accuracy of soil sampling: the spatial distribution of *B. pseudomallei* in soil of a specified sampling site. Soil sampling was performed using a fixed-interval grid of 100 sampling points in each of two sites (disused land and rice field) in northeast Thailand, and the presence and amount of *B. pseudomallei* determined using culture. Mapping of the presence and *B. pseudomallei* count demonstrated that samples taken from areas adjacent to sampling points that were culture positive (negative) for *B. pseudomallei* were also likely to be culture positive (negative), and samples taken from areas adjacent to sampling points with a high (low) *B. pseudomallei* count were also likely to yield a high (low) count (spatial autocorrelation). These data were used as the basis for highlighting several pitfalls in current approaches to soil sampling, together with a discussion of the suitability of a range of sampling strategies in different geographical locations and for different study objectives.

## Introduction

The Gram-negative bacterium *Burkholderia pseudomallei* is the cause of melioidosis and a category B select agent. This organism is present in soil and water across much of southeast Asia and in northern Australia and is increasingly being detected elsewhere, including areas of South America [1]. Melioidosis occurs as a result of exposure to environments containing *B. pseudomallei*. The route of infection is likely to be through direct skin inoculation or contamination of wounds, and more rarely by inhalation and ingestion [2]. Environmental sampling has been widely used to determine the presence of *B. pseudomallei* in an effort to identify geographical distribution of the organism and related risk of infection to humans and livestock [3]–[12]. *B. pseudomallei* has also been sampled from the environment to define the population genetic structure of the organism, to compare this with isolates associated with disease, and during outbreak investigations [13]–[16].

Despite its importance as a cause of natural disease and a bio-threat agent, there is limited information on which to base sampling strategies for *B. pseudomallei* that minimize the false negative rate. Common soil sampling strategies for *B. pseudomallei* are to randomly select multiple sites, and then randomly select one to seven sampling points in each site to collect the soil and test for presence of the organism [3]–[16]. However, it is unclear whether one to seven sampling points are sufficient to detect the presence of the organism in a site. In addition, the optimal distance between sampling points has not been defined.

The semivariogram is a geostatistical tool for determination of the range over which measurements of soil properties are related [17]. The semivariogram has been used to define the range over which counts of specific environmental bacteria are related, and has been reported to range from micrometers to several meters [18]–[20]. Range of spatial autocorrelation is required to determine the optimal distance between sampling points (sampling grid size) [21], [22]. However, no information has been published on the spatial autocorrelation of *B. pseudomallei* over distance between two sampling points (lag distance). The aim of this study was to apply the semivariogram to datasets from two large environmental sampling studies, which previously defined the presence and quantitation of *B. pseudomallei* in disused land [23] and a rice field [24] in nearby regions of northeast Thailand. These data were used as a basis on which to define appropriate environmental sampling strategies for the presence of *B. pseudomallei*.

## Materials and Methods

### Study sites

Soil samples were collected from two locations situated in a rural rice-growing region in Ubon Ratchathani province, northeast Thailand. Sampling of an area of disused land situated to one side of road 231 in Amphoe Meung was performed in September 2005 (the rainy season), as previously described [23]. The long-term history of this land is unknown but its position along the side of a tarmac road which had been in existence in its present form for at least 10 years together with the lack of any signs of recent cultivation suggest that the land had been disused for at least a decade. A member of the study team (GW) who has used the road for more than 10 years confirmed that this land has not been cultivated during this period. The site ran parallel to the road with a brick wall forming the distal boundary. The land plot size was 100 m×25 m and was covered with low-lying scrub. Sampling of an area of rice field situated in Amphoe Lao Sua Kok (a distance of 19 km southwest from the disused land) was performed in May 2007 (start of the rainy season), as previously described [24]. The rice field was several acres in size and has been used for rice cultivation for more than 25 years. The field was divided by raised earth walkways; the area selected was 25 m×25 m in size, and was isolated by raised earth walkways on three sides and by a dirt road on the fourth side. Both sites were wet but not flooded at the time of sampling. The soil type was sandy loam in both sites.

### Sampling strategy

The disused land site was rectangular and a rectangular experimental grid was located at its center. A grid comprising 5×20 plots placed 2.5 m apart on the vertical axis and 1.25 m apart on the horizontal axis (312.5 m^2^) was marked out using string and wooden stakes. Plots were referenced using letters (A to E for horizontal rows viewed with back against the road, row A lying closest to the wall), and numbers (1 to 20 from left to right on the vertical axis). A square experimental grid was used in the rice field. A grid comprising 10×10 plots each measuring 2.5 m by 2.5 m (625 m^2^) was marked out using string and wooden stakes. Sampling plots were also referenced using letters (A to J) for horizontal rows as viewed with back against the earth walkway, row A lying closet to the dirt road) and numbers (1 to 10 from left to right). Soil sampling was performed at the center of each plot. A standard soil sampling technique was performed, as previously described [23], [24]. A hole was dug using a clean spade to a depth of approximately 30 centimeters. A clean plastic bag was placed on weighing scales and a sample of soil (100 grams) was removed from the base of the hole. The spade was cleaned with alcohol before and after sampling at each point.

### Soil culture and *B. pseudomallei* identification and quantitation

Soil was cultured for the presence of *B. pseudomallei* [23], [24]. Colonies of *B. pseudomallei* were initially identified on the basis of colony morphotype [25]. Colonies suspected to be *B. pseudomallei* were tested using the oxidase test, and positive colonies confirmed as *B. pseudomallei* using a highly specific latex agglutination test (positive for *B. pseudomallei* but negative for *B. thailandensis*) [26]. Following confirmation of bacterial identity, colonies with an identical morphotype on a given agar plate were considered to represent *B. pseudomallei*, and a colony count performed to allow calculation of the number of *B. pseudomallei* colonies per gram of soil at each sampling point. The lower and upper limit of detection of the methodology were 1 to ≥10,000 CFU/gm soil, respectively. Proportions of positivity were compared by the Chi-square test and bacterial counts were compared using the Wilcoxon-Mann-Whitney test.

### Analysis of spatial autocorrelation

**Figure 1:**
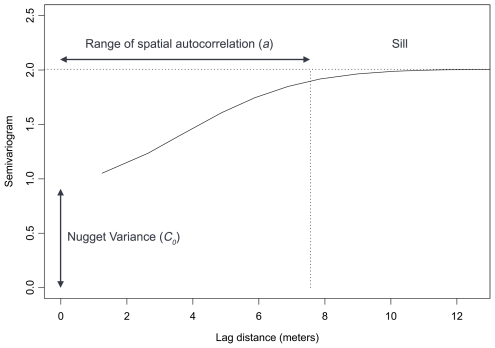
Theoretical semivariogram showing an increase in variance as lag distance increases within *range (a)* (spatial autocorrelation), and upper asymptote (*sill*) as lag distance exceeds the range (no spatial autocorrelation). The *nugget* variance (*C_0_*) exhibits a positive variance at lag distance 0.

Spatial autocorrelation was analysed to quantify the relationship between *B. pseudomallei* count and lag distance between sampling points. To reduce the effect of skew, log10 transformation after adding one to the number of *B. pseudomallei* count was performed. The formula used for the semivariogram was defined as one half the average of the squared difference of log CFU count between all pairs of observations that are the distance *h* apart [27], as follows:
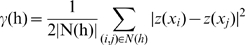
where γ(*h*) is a semivariogram at lag distance of *h*, N(*h*) is the total number of sampling point pairs that are a lag distance apart of *h*, and z(*x_i_*) is log CFU count at location *x_i_*. Figure 1 demonstrates a theoretical semivariogram. The line of the theoretical semivariogram starts from a positive variance if sampling has been repeatedly performed at the same point (where lag distance equals zero). This is known as *nugget* variance, which could be caused by measurement error, random variation or undefined spatial autocorrelation over distances less than the smallest sampling interval. The semivariogram generally rises to an upper asymptote called the *sill*. The sill indicates that the variance at this level is stable and not affected by spatial autocorrelation. The lag distance at which this occurs is called the *range of spatial autocorrelation* or the limit of spatial dependence.

The semivariogram was modelled using the Gaussian equation [27], as follows:
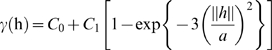
where *C_0_* is a parameter quantifying the nugget effect, *C_1_* is a spatially structured component of the model, and *a* is the range of spatial autocorrelation. The Gaussian model was fitted with non-linear least square regressions.

The semivariogram requires assumptions of stationarity and normality. Stationarity is the condition that mean and variance do not vary significantly in space. Exploratory spatial data analysis (ESDA) described by Cressie [17] was used to evaluate stationarity of log *B. pseudomallei* counts in both fields. The assumption of normality was rarely held because most sampling points in the rice field were culture negative for *B. pseudomallei*. The indicator semivariogram, a robust method of semivariogram, was also used [27]. This was computed by substituting z(*x_i_*) with a binary variable (1 if culture was positive and 0 if culture was negative). The indicator semivariogram was then rescaled by the indicator variance. Semivariograms and indicator semivariograms were also computed for different directions in order to determine whether the pattern of spatial variability changed with direction in the field.

The nugget/sill ratio was used to determine nugget effect on overall variability. A value approaching 1.0 indicates that a large degree of the variability is associated with within sample measurements, and that relatedness between spatially separated measurements is limited. A value close to zero indicates that the relatedness of spatially separated measurements within the range is strong.

All analyses were calculated using Stata 9.0 (College Station, Texas, United States) and S-plus 6.0 with Spatial Stats module (Insightful Corp, Seattle, United States).

## Results

### Presence and quantity of *B. pseudomallei* in two sampling sites

A total of 80 (80%) sampling points in the disused land site were culture positive for *B. pseudomallei*, compared with 28 (28%) positive points in the rice field site (p<0.001, Chi-square test). The median *B. pseudomallei* count for the disused land was 378 cfu/gram soil (range 1 to >10,000, interquartile range (IQR) 55 to 1119), while the median count for the rice field was 700 cfu/gram soil (range 10 to >10,000, IQR 50 to 2810). There was no difference in *B. pseudomallei* count in the positive points of the two sites (p = 0.17, Mann-Whitney test). Further statistics relating to log *B. pseudomallei* counts are available in Table S1. *B. thailandensis* was not detected in either field.

### Mapping of *B. pseudomallei* distribution

**Figure 2:**
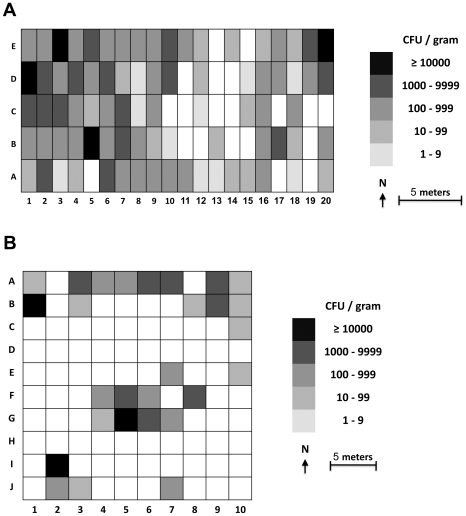
Presence and count (log cfu/gm soil) of *B. pseudomallei* in 100 spaced sampling plots within 312.5 m^2^ of the disused land (2A) and 625 m^2^ of the rice field (2B). The gray scale represents the amount of *B. pseudomallei* in soil, with pure white indicating a point negative for *B. pseudomallei*.

Mapping of log *B. pseudomallei* counts showed that sampling plots with high (low) *B. pseudomallei* count were likely to be surrounded by sampling points with high (low) count (Figure 2). The highest density of *B. pseudomallei* (>10,000 CFU/gram) was similar in both fields. Stationarity of log *B. pseudomallei* counts was tested; no major trends of mean and variance were observed with direction in either field. Therefore, correlation (semivariogram) between any two locations depended only on the lag distance between them, not their exact locations.

### Spatial autocorrelation

**Figure 3:**
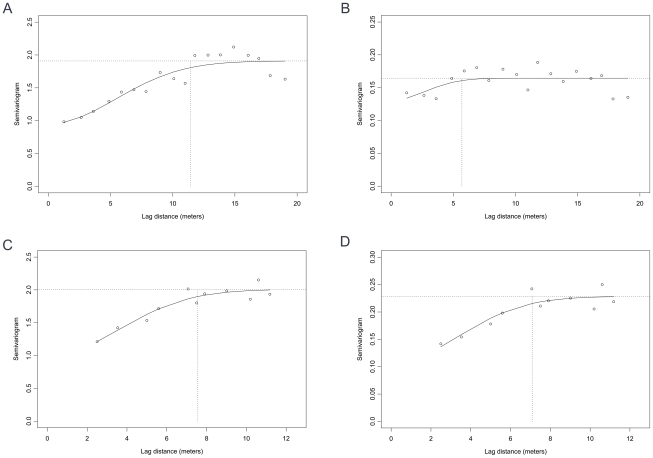
Semivariograms for count (log cfu/gm soil) of *B. pseudomallei* and indicator semivariograms for presence of *B. pseudomallei* over the lag distance (in meters). The solid line represents the fitted Gaussian model. In the disused land (3A and 3B), *range* of spatial autocorrelation for log CFU count was 11.4 meters, and *range* of spatial autocorrelation for presence of the organism was 5.7 meters. In the rice field (3C and 3D), *range* of spatial autocorrelation for log CFU count was 7.6 meters, and *range* of spatial autocorrelation for presence of the organism was 7.1 meters.

Spatial autocorrelation of log *B. pseudomallei* counts in the disused land and the rice field was present for up to 11.4 meters and 7.6 meters of lag distance, respectively (Table 1, Figure 3). The nugget/sill ratios were 0.50 and 0.49 for the disused land and the rice field, respectively, indicating a moderate degree of spatial autocorrelation of log CFU count at both sites. Using a robust method (indicator semivariogram), we found that the range of spatial autocorrelation for presence of *B. pseudomallei* in the rice field was 7.1 meters, which was not different from the range of spatial autocorrelation for log *B. pseudomallei* counts (7.6 meters). However, in the disused land where the proportion of positive points was very high, spatial autocorrelation for presence of *B. pseudomallei* was lower (nugget/sill ratio 0.79), indicating that variability of test positivity in this field was mainly caused by measurement error or random variation. A directional semivariogram and directional indicator semivariogram showed that the patterns of spatial variability were not different with direction in either field (Figure S1 and S2).

**Table 1:** Summary of parameters obtained from fitting a Guassian equation to the semivariograms and indicator semivariograms.

Field	Model	Direction	Range (m)	Nugget (*C_0_*)	Sill (*C*)	Nugget effect (*C_0_/C*)
Disused land	Semivariogram	Omnidirectional	11.4	0.95	1.91	0.50
		N45E	9.3	0.90	1.79	0.51
		N45W	13.7	0.99	2.07	0.48
	Indicator semivariogram	Omnidirectional	5.7	0.13	0.16	0.79
		N45E	6.3	0.13	0.17	0.75
		N45W	5.5	0.13	0.17	0.73
Rice field	Semivariogram	Omnidirectional	7.6	0.99	2.01	0.49
		N45E	8.0	0.96	2.09	0.46
		N45W	7.0	1.02	1.92	0.53
	Indicator semivariogram	Omnidirectional	7.1	0.11	0.23	0.47
		N45E	7.2	0.10	0.23	0.46
		N45W	7.0	0.11	0.23	0.48

### Sampling strategy

**Figure 4:**
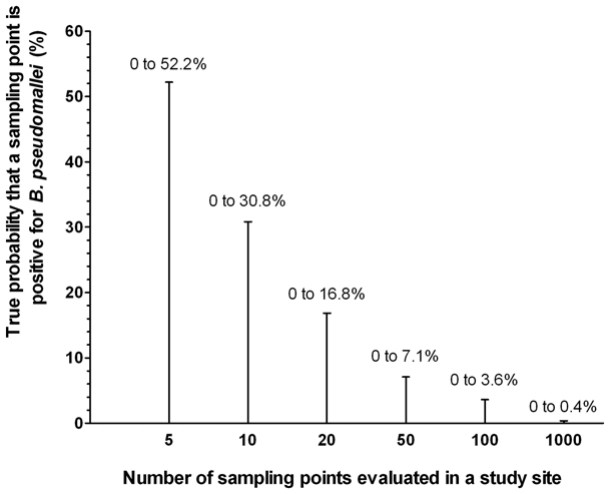
Estimated 95% confidence intervals (log scale) plotted against the number of soil samples taken for a given test site. Confidence in a negative result is related to the number of samples taken and is very low for a small sample size. Footnote to Figure 4. Given that the true probability of the organism being in the evaluated rice field is 28%, it is still not uncommon that random sampling of 10 independent points would yield all negative results as 95%CI of 10 sampling points includes the probability of 28%.

Findings from our data were combined with a statistical approach to define common pitfalls of sampling for the detection of *B. pseudomallei* in soil. The presence of *B. pseudomallei* in the soil is hazardous and of the potential errors associated with soil sampling, we considered a false negative result as the one of greatest significance. This would result in under-reporting of the geographic distribution of the organism, and could result in the dissemination of inaccurate and misleading information in areas defined as false negative. In light of this, we considered the reliability of a negative culture result in a given study site based on the 95% binomial confidence interval (CI) (Figure 4). For example, if only 10 independent samples are randomly taken in one area and all are negative, the exact 95% binomial CI ranges from 0 to 30.8%, which means that even if the probability of sampling points positive for *B. pseudomallei* is actually as high as 28%, it is still not uncommon for all 10 independent samples randomly selected from this site to be negative (as the 95%CI includes 28%). If 100 samples are taken, the exact 95% binomial CI is 0 to 3.6%, and if 1000 samples are taken the CI is 0 to 0.4%, and so on (Figure 4). The number of sampling points taken and distance between them should be based on a formal sample size calculation [21], [22]. The semivariograms and ranges of spatial autocorrelation defined in this study allowed us to determine the optimal distance between sampling plots in this geographical area [21], [22]. The optimal distance between sampling points has been described as being half the range of the spatial autocorrelation observed in the semivariogram [22]. As the range of spatial autocorrelation for the presence of *B. pseudomallei* were 5.7 and 7.1 meters of lag distance, for northeast Thailand we propose that the initial sampling grid size during future studies could be between 2.5 and 3.5 meters.

## Discussion

### Spatial autocorrelation of *B. pseudomallei* in soil

The aim of this study was to define the spatial distribution *B. pseudomallei* in soil using a geostatistical approach to analyse datasets from two large environmental sampling studies. We found that *B. pseudomallei* was not uniformly distributed in the soil, but rather was randomly distributed with spatial autocorrelation. Samples taken from areas adjacent to sampling points with a high (low) *B. pseudomallei* count were also likely to yield a high (low) count. This finding is consistent with previous studies of other microorganisms present in soil [18]–[20]. Several explanations have been proposed for this phenomenon. Bacterial communities may be affected by an uneven distribution of organic matter from which soluble compounds are diffusing, with bacterial density greatest close to the organic matter [28]. Another possibility is that the spatial pattern may reflect an effect of regulation within the bacterial community itself, in which release of specific bacterial factors involved in bacterial gene regulation influences growth within the community. This is supported by a growing body of evidence that bacterial quorum-sensing occurs in soil [29]–[31].

### Difference in positivity rate and spatial relationship observed between rice field and disused land

This study also demonstrated a marked difference in the proportion of samples positive for *B. pseudomallei* in the disused land site versus the rice field site, despite the observation that the *B. pseudomallei* counts per gram of soil at positive spots were not different between the two sites. This was reflected in the shorter range of spatial autocorrelation for log CFU count in the rice field compared with the disused land (7.6 meters versus 11.4 meters). Rice fields undergo repeated flooding, ploughing, planting, rice stubble burning and the application of chemical fertilizers and pesticides. *B. pseudomallei* may also be influenced by the presence of rice. A difference in bacterial communities present in rice field and disused land has been described previously in regions where *B. pseudomallei* was not present, and agricultural practices have been reported to lead to reversible changes in the community structure of environmental *Burkholderia* species other than *B. pseudomallei* [32]–[34]. However, this finding should not be interpreted to mean that the proportion of *B. pseudomallei* in disused land is higher than in rice fields because the number of sites sampled is low and the sampling points within each field were correlated. Intensive sampling to determine the distribution of *B. pseudomallei* in a field has not been subjected previously to systematic study. Future studies are required to investigate whether these findings are reproducible.

### Soil sampling strategies

The best soil sampling strategy should have adequate power to detect the presence of *B. pseudomallei* in a field, regardless of whether the prevalence of the organism is high or low. Our study was based on a fixed-interval sampling strategy which was used for its simplicity, and because this is a recommended strategy for the generation of semivariograms [21], [35]. Alternative sampling strategies have been described, including random sampling, stratified sampling, adaptive sampling and multistage sampling [36]. The most appropriate sampling strategy will depend on the objectives of the study, and whether any information is already available for the geographical area to be sampled. In stratified sampling, the experimental area is divided into zones or strata and unequal numbers of samples are taken from each stratum. Stratified sampling may be used where sampling areas differ greatly or prior information indicates that the *B. pseudomallei* prevalence varies across the study area. We showed that sampling less than 10 sampling points per site may give a false negative result, even if the actual probability that an independent sampling point will be positive is as high as 28%. Adaptive sampling may be a suitable approach for the detection of *B. pseudomallei* in an area where the presence and/or distribution of *B. pseudomallei* is unknown. For example, a pilot study could be performed in a defined experimental area in which 20 random points are sampled and tested for the presence of *B. pseudomallei*. If any sampling point is positive for *B. pseudomallei*, this confirms the presence of the organism and therefore an area of risk to humans and livestock. If no sampling points are positive for *B. pseudomallei*, a second round of sampling is done in which a larger number of random points or all possible sampling grid points are sampled in the same area. Based on our analysis, we recommend that a minimum of 100 sampling points for an area of land measuring 30 m×30 m should be taken during stage 2 in the event that the first round of sampling is negative.

Random sampling with ad hoc strategies have been used previously to define the presence of *B. pseudomallei* throughout southeast Asia and northern Australia [3]–[16]. The methodology for sampling including calculations or justification of sample size was not usually specified, and the number and position of sampling points were probably selected on an ad hoc basis. This has provided important information on geographical areas of positivity and has shown that *B. pseudomallei* load is higher in areas of south Asia including northeast Thailand and Laos than in northern Australia. However, the sampling strategies such as those previously used in which a small number of sampling points were tested per field and/or distances between the points were short could potentially underestimate the geographical distribution of positive sites, particularly in an area of low prevalence.

We propose that a multilevel approach is suitable to determine the geographical distribution of *B. pseudomallei* within an endemic region such as the province of Ubon Ratchathani. This sampling method involves considering a primary sample unit such as field, and a secondary sample unit such as sampling point in each field. Defined areas of land (the primary sample unit) are selected from the entire region using a sample size calculation. Each experimental area is then sampled using an adequate number of sampling points using an initial sampling grid size (in northeast Thailand) of 2.5 to 3.5 meters (for example, 100 sampling points in a single site measuring 30 m×30 m) or based on new data generated from future studies. Sampling strategies in each field could be based on random sampling, fixed-interval sampling, stratified sampling or adaptive sampling, as described above. This extensive dataset would give a broad insight into the distribution of *B. pseudomallei* across the region.

The optimal sampling grid size should be calculated based on the spatial autocorrelation (semivariogram) of the presence of organism in a given area and the level of precision required [21], [22]. Variability in *B. pseudomallei* count in soil within and between different countries is well described [4], [10], [11], and the proposed sampling distance may not hold true in areas where the predicted *B. pseudomallei* count in soil is markedly different from that found in this study, since the range of autocorrelation is likely to differ [18]. To evaluate the range of spatial autocorrelation in different geographic areas, we suggest that a semivariogram and area-specific spatial autocorrelation values be calculated from initial sampling of at least 100 to 150 sampling points [35].

### Limitations and future studies

The moderate degree of nugget sill ratio found in this study could be due to variation in the soil sampling technique used. The standard methodology for culture of *B. pseudomallei* from soil involved the addition of water to the soil sample followed by vigorous manual mixing and overnight sedimentation, after which the supernatant was removed for culture [4]–[6], [8]–[10], [13]–[16]. It is possible that *B. pseudomallei* may either replicate or die during the overnight sample preparation, and some strains may not grow in the culture medium. Molecular detection techniques such as PCR may resolve this problem. DNA extraction from soil followed by detection of *B. pseudomallei* by real-time PCR has been reported to be more sensitive than culture [11], and further studies are required in which PCR is evaluated in a range of geographical settings. The possibility that soil from multiple sampling points in a field could be bulked and DNA extracted and tested as a single sample would allow for more rapid coverage of a potentially large sample size [34]. It is likely, however, that culture will be used in the immediate future to determine the presence of *B. pseudomallei* in the environment. Further studies are now required to extend our understanding of the global distribution of *B. pseudomallei* in the environment, together with an evaluation of factors such as the physical properties of soil and the effect of vegetation that influence its spatial distribution.

### Concluding comments

This is the first published study to define spatial distribution of *B. pseudomallei* in the environment. Our data are likely to be specific to northeast Thailand and our focus was primarily the issue of false negatives. As such, it does not attempt to determine optimal strategies necessary for all applications, such as the strategy necessary to obtain an unbiased snapshot of bacterial population genetic structure. However, our findings have major implications for future environmental studies of *B. pseudomallei*, and highlight both the critical importance of study design and methodological approach and the need for further studies in this area.

## Supporting Information

Figure S1 Directional semivariograms for quantitation of *B. pseudomallei* (log cfu/gram soil) over the lag distance (in meters) in the disused land at N45E (1A) and N45W (1B), and in the rice field at N45E (1C) and N45W (1D).
(0.43 MB TIF)

Figure S2 Directional indicator semivariograms for presence of *B. pseudomallei* over the lag distance (in meters) in the disused land at N45E (1A) and N45W (1B), and in the rice field at N45E (1C) and N45W (1D).
(0.43 MB TIF)

Table S1 Summary statistics for quantitative *B. pseudomallei* data from the disused land and the rice field in log cfu/gram of soil.
(0.04 MB DOC)
