# Supplementary figures and images for: Burkholderia pseudomallei Is Spatially Distributed in Soil in Northeast Thailand
Source: PLoS Negl Trop Dis. 2010 Jun 1;4(6):e694. doi: 10.1371/journal.pntd.0000694 (PMC2879387; doi:10.1371/journal.pntd.0000694)

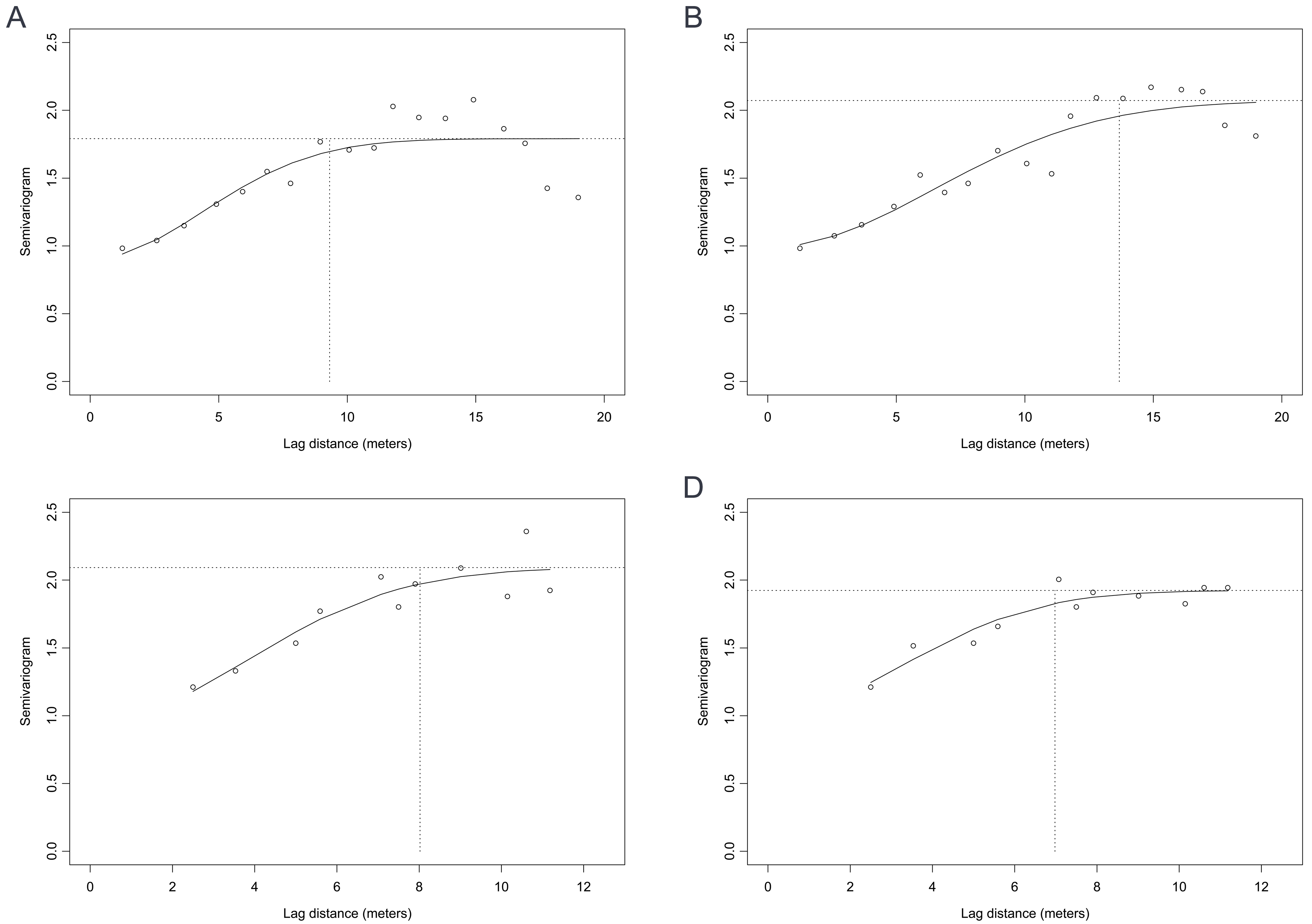

Supplement: Figure S1 — Directional semivariograms for quantitation of B. pseudomallei (log cfu/gram soil) over the lag distance (in meters) in the disused land at N45E (1A) and N45W (1B), and in the rice field at N45E (1C) and N45W (1D). (0.43 MB TIF) [file pntd.0000694.s001.tif]

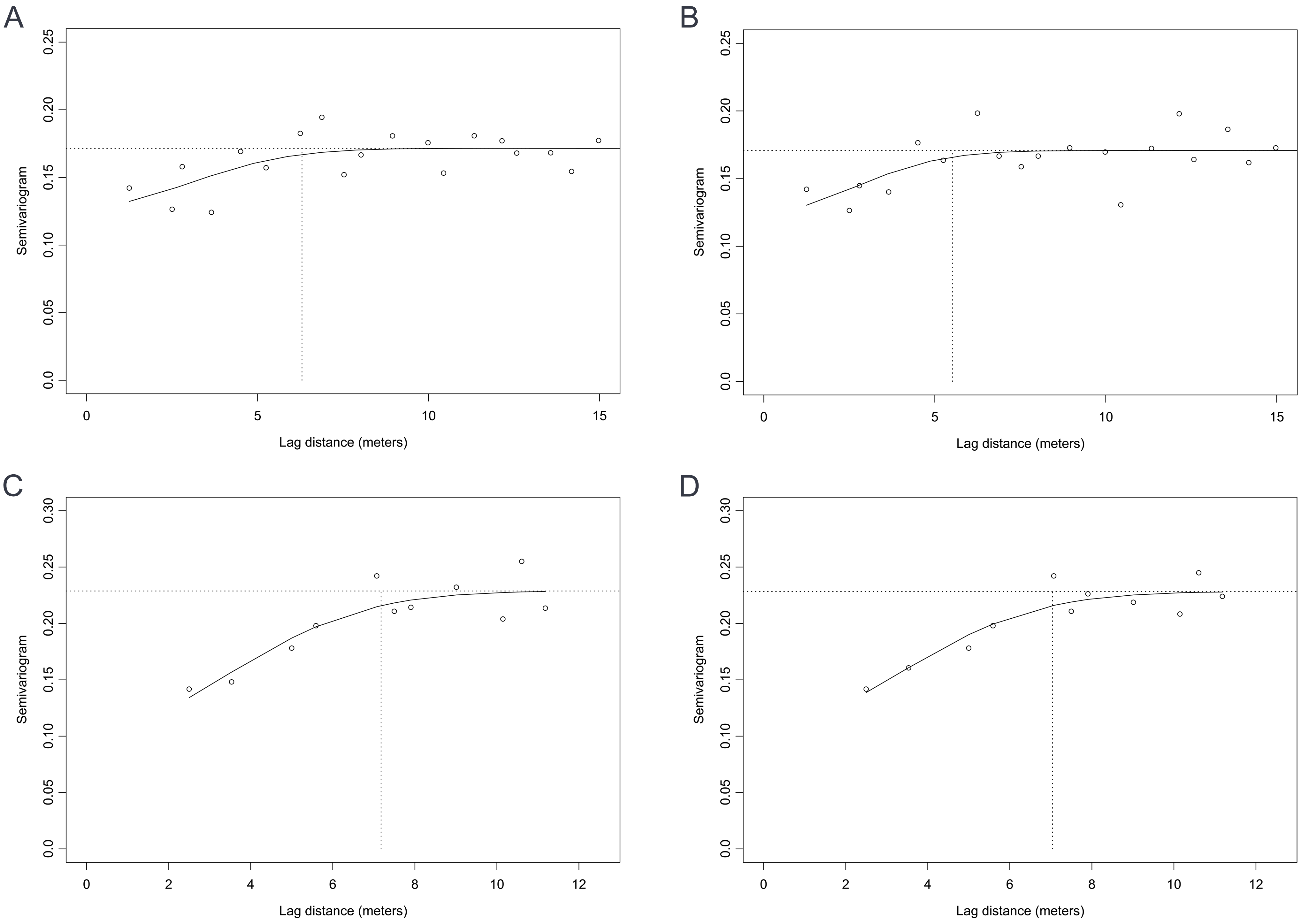

Supplement: Figure S2 — Directional indicator semivariograms for presence of B. pseudomallei over the lag distance (in meters) in the disused land at N45E (1A) and N45W (1B), and in the rice field at N45E (1C) and N45W (1D). (0.43 MB TIF) [file pntd.0000694.s002.tif]
