# Supplementary material for: Burkholderia pseudomallei Is Spatially Distributed in Soil in Northeast Thailand
Source: PLoS Negl Trop Dis. 2010 Jun 1;4(6):e694. doi: 10.1371/journal.pntd.0000694 (PMC2879387; doi:10.1371/journal.pntd.0000694)
Supplement: Table S1 — Summary statistics for quantitative B. pseudomallei data from the disused land and the rice field in log cfu/gram of soil. (0.04 MB DOC) [file pntd.0000694.s003.doc]

**Table S1.** Summary statistics for quantitative *B. pseudomallei* data from the disused land and the rice field in log cfu/gram of soil

| Field | Number | Mean | Min | 1^st^ Q | 2^nd^ Q | 3^rd^ Q | Max | SD | Skewness | Kurtosis |
| --- | --- | --- | --- | --- | --- | --- | --- | --- | --- | --- |
| Disused land | 100 | 1.90 | 0 | 0.66 | 2.31 | 2.86 | 4.00 | 1.27 | 0.31 | 1.78 |
| Rice field | 100 | 0.75 | 0 | 0 | 0 | 1.37 | 4.00 | 1.30 | 1.40 | 3.36 |
